# Cardiorespiratory optimal point in post-COVID-19 patients: a cross-sectional study

**DOI:** 10.1590/S1678-9946202466014

**Published:** 2024-02-19

**Authors:** Karinne Simões da Cruz Santos, Gabriela Menezes Gonçalves de Brito, Enaldo Vieira de Melo, Antônio Carlos Sobral Sousa, Paulo Ricardo Martins-Filho, Milena dos Santos Barros Campos

**Affiliations:** 1 Universidade Tiradentes, Departamento de Medicina, Aracaju, Sergipe, Brazil; 2 Universidade Federal de Sergipe, Departamento de Medicina, Aracaju, Sergipe, Brazil; 3 Universidade Federal de Sergipe, Programa de Pós-Graduação em Ciências da Saúde, Aracaju, Sergipe, Brazil; 4 Rede D’Or São Luiz, Clínica e Hospital São Lucas, Aracaju, Sergipe, Brazil; 5 Universidade Federal de Sergipe, Hospital Universitário, Divisão de Cardiologia, Aracaju, Sergipe, Brazil; 6 Universidade Federal de Sergipe, Hospital Universitário, Laboratório de Patologia Investigativa, Aracaju, Sergipe, Brazil

**Keywords:** COVID-19, Post-acute COVID-19 syndrome, Exercise test, Cardiovascular system

## Abstract

The varied clinical presentations of SARS-CoV-2 infection have raised concerns about long-term consequences, especially “long-COVID” or “post-COVID-19 syndrome.” In this context, the cardiorespiratory optimal point (COP) within the Cardiopulmonary Exercise Test (CPET) emerges as a crucial metric for evaluating functional capacities and detecting cardiovascular and pulmonary anomalies post-COVID-19. This study aimed to assess COP values among post-COVID-19 patients and categorized them based on the initial severity of their disease. In this cross-sectional study conducted in the Northeast Brazil, 80 patients (26 females and 54 males) previously infected with SARS-CoV-2 underwent CPET. We clinically stratified patients into mild, moderate, or severe COVID-19 categories and assessed COP values and other cardiorespiratory metrics. We found differences in the predicted COP between patients with mild and severe COVID-19 (p=0.042). Additionally, patients with moderate and severe COVID-19 record had an average COP value exceeding 22. Other parameters, including respiratory exchange ratio, heart rate, and oxygen uptake efficiency slope, did not differ across the groups. Patients with a history of severe COVID-19 showed altered COP values, suggesting potential discrepancies in cardiovascular and respiratory system integration. The outcomes emphasize the importance of continuous monitoring and assessment of the cardiorespiratory domain for post-COVID-19 patients. Further research is needed to understand the relationship between elevated COP in post-severe COVID-19 and its long-term prognostic implications.

## INTRODUCTION

The infection caused by the SARS-CoV-2, which leads to COVID-19, shows a clinical spectrum ranging from asymptomatic cases to severe multisystemic failures. A significant proportion of individuals initially exhibit non-specific symptoms, but in moderate cases, the disease can progress to pulmonary complications. In its severe form, the infection can culminate in acute respiratory distress syndrome^1^.

Emerging data indicate a concerning persistence of symptoms following the acute phase of COVID-19. This condition, known as “long-COVID” or “post-COVID-19 syndrome,” involves persistent sequelae for at least eight weeks, predominantly as musculoskeletal and cardiorespiratory dysfunctions^2,3^. This prolonged morbidity associated with this infection underscores the urgent need for advanced diagnostic tools.

The Cardiopulmonary Exercise Test (CPET) is the most appropriate assessment method to evaluate functional capacities and discriminate cardiovascular and pulmonary anomalies^4^. Within CPET metrics, the cardiorespiratory optimal point (COP) has garnered attention for its ability to illustrate the interplay between respiratory and cardiovascular functions^5^. Researchers have empirically established the prognostic significance of COP, particularly when combined with other CPET metrics^6^.

Given the persistent physical limitations and dyspnea experienced by many post-COVID-19 patients, the diagnostic precision of CPET becomes necessary. Notably, a substantial number of these patients fail to achieve maximal exertion levels during tests, highlighting the importance of metrics such as COP. This study aims to explore COP values in post-COVID-19 patients, stratifying the findings based on the initial severity of the disease.

## MATERIALS AND METHODS

### Study design, sample, and eligibility criteria

This cross-sectional study was carried out at a private cardiology reference hospital in Aracaju city, Sergipe State, Brazil, focusing on patients undergoing CPET. A total of 80 individuals (26 females and 54 males) previously infected with SARS-COV-2 were evaluated. These participants had an average time of 137 days since infection, with a minimum of one month. Participants with a confirmed COVID-19 diagnosis by RT-PCR were included and those with a respiratory quotient of less than 1.0 during CPET were excluded. The study was approved by the institution ethics committee (CAAE Nº 63931122.60000.5371).

### Clinical data collection

Before the tests, detailed anamneses were performed to collect information on comorbidities, lifestyle habits, COVID-19 progression, infection severity, length of hospitalization, and any persisting post-acute phase symptoms. COVID-19 severity was clinically classified into: mild (individuals with any COVID-19 symptoms without dyspnea or reduced oxygen saturation); moderate (respiratory lower tract disease with oxygen saturation ≥ 95% in ambient air), and severe (oxygen saturation < 95% in ambient air, with dyspnea, pulmonary infiltrates > 50%, or acute respiratory syndrome). All participants underwent resting electrocardiograms before CPET.

### Cardiopulmonary exercise test

A graded ramp exercise regimen for CPET was employed, including a 1-min active and 3-min passive recovery phase. Blood pressure was measured using a calibrated aneroid sphygmomanometer. Maximum exercise intensity was determined by the respiratory exchange ratio (RER), specifically when the carbon dioxide production (V̇CO2) to oxygen consumption (V̇O2) ratio exceeded 1.1. Data was collected in 10-s intervals, using the Córtex Metalyser 3B gas analyzer (Micromed) interfaced with Ergo PC Elite (version 3.3.6.2, Micromed^™^, Brazil) software, and ECG signals were collected during exertion with a Micromed digital electrocardiograph. The exercise protocol used the Inbrasport Super ATL treadmill.

Before exertion, preliminary spirometry was conducted, including flow-volume curve evaluation, measurements of forced expiratory volume in one second (FEV1), forced vital capacity (FVC), Tiffeneau-Pinelli Index (FEV1/FVC ratio), and forced expiratory flow at 25^–^75%.

Peak oxygen consumption (V̇O2 peak) was determined as the 30-s ending average and was expressed as mL kg^1^ min^1^. Anaerobic threshold oxygen consumption (V̇O2 AT) was identified via the Vslope graph, illustrating the V̇O2 to V̇CO2 slope, or by ventilatory threshold evaluations and end-expiratory pressures, when required. Oxygen pulse (PO2) was estimated as the V̇O2 to heart rate (HR) ratio. Ventilatory efficiency of oxygen uptake (OUES) was verified with the gradient from the linear relationship of ventilation (V̇E log10) to V̇O2. The V̇E/V̇CO2 slope indicated ventilatory efficiency, with values <35 considered normal.

Finally, COP was determined as the lowest ventilatory oxygen equivalent (V̇E/ V̇O2), assessed minute-by-minute during the exercise phase. Test termination criteria followed the guidelines by the III Brazilian Society of Cardiology on Ergometric Testing^4^.

### Statistical analysis

Categorical variables were presented as frequencies and percentages and continuous variables as mean with standard deviation. A one-way analysis of variance (ANOVA) based on COVID severity was employed, with post hoc comparisons conducted using the Tukey’s method. All reported p-values were two sided and considered significant at a 5% significance level. Data were analyzed using JASP software (version 0.13, JASP Team, Amsterdam, Netherlands).

## RESULTS

The sample’s mean age was 45.8 (13) years. We stratified the patients into three groups based on their COVID-19 severity: mild (n=33), moderate (n=25), and severe (n=22). Males predominated across all groups, accounting for 67.5% of the sample. Patients with moderate COVID-19 were older than those in the other groups.

Regarding cardiorespiratory metrics, the groups showed no differences in the percentage of peak oxygen consumption (V̇O2) relative to the predicted values based on Wasserman’s equation. However, the severe COVID-19 group had a lower percentage of V̇O2 at the anaerobic threshold compared to the mild group (p=0.014). We also found differences in the predicted COP between patients with mild and severe COVID-19 (p=0.042). Additionally, patients with moderate and severe COVID-19 histories had an average COP value exceeding 22. Other parameters, such as RER, maximum HR, V̇E/V̇CO2 slope, PO2, and OUES, showed no differences across groups (Table 1).

**Table 1:** Clinical, demographic, and cardiopulmonary exercise test parameters by COVID-19 severity.

Parameter	COVID-19 severity			p-value

	Mild (n=33)	Moderate (n=25)	Severe (n=22)	
**Clinical and demographic**				
Males	22	14	18	0.216
Age (years)	44.3 ± 12.1*	52.5 ± 11.3*	44.7 ± 13.6	0.025
Time since COVID-19 (days)	134.9 ± 82.5	117.0 ± 87.1	156.1 ± 102.8	0.340
**CPET**				
Maximum HR (bpm)	94.7 ± 10.6	100.0 ± 14.8	102.0 ± 29.6	0.319
RER	1.1 ± 0.1	1.1 ± 0.1	1.1 ± 0.1	0.128
Percent-predicted peak V̇O2	79.2 ± 16.5	77.0 ±12.2	71.2 ±14.8	0.182
Predicted V̇O2 AT (ml.kg^-1^. min^-1^)	18.4 ± 5.7*	13.7 ± 3.6*	15.0 ± 4.1	0.001
Percent-predicted V̇O2 AT	60.0 ± 8.2*	57.8 ± 10.1	52.7 ± 8.6*	0.014
Percent-predicted COP	92.6 ± 12.9*	93.5 ± 10.4	101.6 ± 17.1*	0.042
COP	21.5 ± 2.7	23.0 ± 2.6	22.8 ± 3.4	0.104
Percent-predicted PO2	81.9 ± 15.9	82.3 ± 10.2	79.4 ± 15.5	0.820
V̇E/V̇CO2 slope	31.9 ± 3.4	33.8 ± 3.9	33.6 ± 5.6	0.216
OUES (L.min^−1^)	2.3 ± 1.0	2.0 ± 0.7	2.5 ± 0.8	0.288

HR = heart rate; RER = respiratory exchange ratio; V̇O2 peak = peak oxygen uptake; AT = anaerobic threshold; CPET = cardiopulmonary exercise test, COP = cardiorespiratory optimal point, PO2 = oxygen pulse, V̇E/V̇CO2 slope = ventilatory efficiency, OUES = oxygen uptake efficiency slope; *statistically significant difference between groups.

## DISCUSSION

Our study indicates a potential association between increased COP values and COVID-19 severity, with individuals who experienced severe COVID-19 showing higher COP values compared to those with milder symptoms. This observation suggests that patients who have had severe COVID-19 and are experiencing post-COVID symptoms may show signs of impaired coordination between their cardiovascular and respiratory systems. This potential alteration in COP values calls for more in-depth research, considering the intricate cardiovascular issues frequently linked with post-COVID conditions.

Emerging evidence suggests that even mild COVID-19 infections can have a lasting impact on the cardiovascular system. Many patients show myocardial inflammation, ventricular dysfunction, and vascular complications weeks to months after recovery^7-9^. Moreover, an increased prothrombotic state in post-COVID-19 patients raises concerns about long-term thromboembolic event risks^10,11^. These cardiovascular complications underscore the need for long-term monitoring and comprehensive assessment, especially in patients with elevated COP values.

Research indicates that post-COVID-19 individuals experience a range of physical symptoms, including weakness, muscle pain, and respiratory symptoms^12^. Fatigue, reported as one of the most persistent symptoms, affects a large percentage of individuals even more than a year post-infection^13^. Dyspnea is also frequently reported and is associated with poorer quality of life, decreased functional status, and increased healthcare need^14^. These symptoms can persist beyond the acute phase and are not limited to individuals with severe COVID-19. Recent findings attribute the long-term impact of these symptoms to post-viral muscular deconditioning syndrome, where the virus aftermath severely limits one’s exercise capacity^15,16^.

Cardiorespiratory fitness is a proven robust marker of functional capacity, cardiovascular health, and overall mortality. However, COP introduces a novel metric within submaximal exercise testing, offering an additional dimension to cardiovascular risk assessment. Recent studies emphasize COP association with coronary disease risk, cardiovascular diseases, sudden cardiac death, and all-cause mortality, underscoring its clinical and prognostic relevance in CPET assessments^17,18^. Additionally, the cardiovascular effects of COVID-19, such as myocarditis, arrhythmias, and heart failure, highlight the need for comprehensive cardiac assessment tools like CPET post-infection.

## CONCLUSION

In summary, our study underscores the increased COP in patients with severe COVID-19 compared to those with milder symptoms. Despite its significance, this study had limitations, including the reliance on convenience sampling and a single-time CPET evaluation. Further research is crucial to establish the relationship between elevated COP after severe COVID-19 and long-term prognosis.
